# Health outcomes from a 12‐week healthy body and mind program for people with osteoarthritis and thinking concerns/dementia: A pilot randomised control trial

**DOI:** 10.1002/alz70860_101878

**Published:** 2025-12-23

**Authors:** Claire V Burley, Matthew D Jones, Nattai Borges, Henry Brodaty, Belinda J Parmenter

**Affiliations:** ^1^ School of Health Sciences, University of New South Wales, Sydney, NSW, Australia; ^2^ Dementia Centre of Excellence, Curtin University, Perth, Western Australia, Australia; ^3^ Centre for Healthy Brain Ageing, University of New South Wales, Sydney, NSW, Australia; ^4^ Department of Exercise Physiology, School of Health Sciences, University of New South Wales, Sydney, NSW, Australia; ^5^ Centre for Pain IMPACT, Neuroscience Research Australia, University of New South Wales, Sydney, NSW, Australia; ^6^ Centre for Healthy Brain Ageing (CHeBA), UNSW Sydney, Sydney, NSW, Australia; ^7^ School of Health, University of the Sunshine Coast, Sippy Downs, QLD, Australia

## Abstract

**Background:**

Osteoarthritis (OA) has been reported in approximately1‐in‐2 older adults and is associated with a 1.5‐fold increase in dementia risk. Half of overall disease burden is due to modifiable factors such as physical activity and lifestyle. Currently no programs exist for people living with OA and thinking concerns/dementia. The aims of this pilot randomised controlled trial (RCT) are to determine whether a 12‐week multidisciplinary *Healthy Body & Mind Program* improves health outcomes in people living with OA and thinking concerns/dementia.

**Method:**

Eighteen consenting participants with OA and thinking concerns/dementia (mean age=71.5±5.7years; mean telephone Montreal Cognitive Assessment [MoCA]=18.9±2.1) were randomly allocated to the 12‐week program or usual medical care (wait‐list control). Outcome measures included quality of life (QoL) [SF‐36], pain (Western Ontario and McMaster Universities Arthritis Index [WOMAC]), cognition (MoCA and Cambridge Cognition multitasking test [MTT], spatial working memory [SWM] and paired associated learning [PAL]) and psychological health (Depression, Anxiety and Stress Scale [DASS‐21]). Data were analysed using ANOVAs (intervention/control) and paired t‐tests (baseline/follow‐up). Cognition data were available for baseline/follow‐up only.

**Results:**

Repeated measures ANOVAs revealed significant time by group interactions for overall pain (*p* = .034;η^2^=0.348) and QoL scores (*p* = .025;*η* ^2^=0.380), where outcomes improved for the intervention group (*N* = 9) and worsened for the control group (*N* = 4). Significant interactions were also observed for the QoL subscales emotional (*p* = .036;*η* ^2^=0.342), social (*p* < .001;*η* ^2^=0.645), and general health (*p* = .046;*η* ^2^=0.046). No significant differences were observed for psychological health measures. A clinically meaningful improvement was observed in overall cognition (mean MoCA increased by 1.2 points). T‐tests revealed significant improvements in the MTT (including number of incongruent errors (*p* = .03;d=0.84), mean reaction latency on congruent trials (*p* = 0.008;d=17.07) and incongruent trials (*p* = .01;d=5.14), mean multitasking cost (*p* = 0.009;d=1.80), total correct responses (*p* = .01;d=3.39) and total errors (*p* <0.005;d=1.57). No significant differences were observed for SWM or PAL.

**Conclusion:**

In this pilot 12‐week RCT, the *Healthy Body & Mind Program* improved pain, QoL, and cognition in people living with OA and thinking concerns or dementia. Future research should scale‐up and deliver this program to larger populations to identify if such programs have the potential to improve public health and reduce dementia risk at a national level.

